# Three-Dimensional Pseudo-Continuous Arterial Spin Labeling Parameters Distinguish Pediatric Medulloblastoma and Pilocytic Astrocytoma

**DOI:** 10.3389/fped.2020.598190

**Published:** 2021-03-02

**Authors:** Nguyen Minh Duc

**Affiliations:** ^1^Doctoral Program, Department of Radiology, Hanoi Medical University, Ha Noi, Vietnam; ^2^Department of Radiology, Pham Ngoc Thach University of Medicine, Ho Chi Minh City, Vietnam; ^3^Department of Radiology, Children's Hospital 02, Ho Chi Minh City, Vietnam

**Keywords:** posterior fossa tumor, magnetic resonance imaging, pilocytic astrocytoma, medulloblastoma, three-dimensional pseudo-continuous arterial spin labeling

## Abstract

**Introduction:** Arterial Spin Labeling (ASL), a perfusion assessment without using gadolinium-based contrast agents, is outstandingly advantageous for pediatric patients. The differentiation of medulloblastomas from pilocytic astrocytomas in children plays a significant role in determining treatment strategies and prognosis. This study aimed to assess the use of ASL parameters during the differentiation between pediatric medulloblastoma and pilocytic astrocytoma.

**Methods:** The institutional review board of Children's Hospital 2 approved this prospective study. The brain magnetic resonance imaging (MRI) protocol, including axial three-dimensional (3D) pseudo-continuous ASL, was evaluated in 33 patients, who were divided into a medulloblastoma group (*n* = 25) and a pilocytic astrocytoma group (*n* = 8). The quantified region of interest (ROI) values for the tumors and the tumor to parenchyma ratios were collected and compared between the two groups. Receiver operating characteristic (ROC) curve analysis and the Youden index were utilized to identify the best cut-off, sensitivity, specificity, and area under the curve (AUC) values for significant ASL parameters.

**Results:** The cerebral blood flow (CBF) and the ratio between the CBF of the tumor relative to that of the parenchyma (rCBF) values for medulloblastomas were significantly higher than those for pilocytic astrocytomas (*p* < 0.05). A cut-off value of 0.51 for rCBF was able to discriminate between medulloblastoma and pilocytic astrocytoma, generating a sensitivity of 88%, a specificity of 75%, and an AUC of 83.5%.

**Conclusion:** The rCBF measurement, obtained during MRI with 3D pseudo-continuous ASL, plays a supplemental role in the differentiation of medulloblastoma from pilocytic astrocytoma.

## Introduction

The second-most prevalent tumor types in children are the central nervous system (CNS) tumors. In contrast to adults, 60% of pediatric intracranial tumors originate in the posterior cranial fossa. Pilocytic astrocytoma and medulloblastoma are the two most common tumors that occur in the posterior fossa (1, 2). Pilocytic astrocytoma is classified as a predominantly World Health Organization (WHO) grade I cystic tumor, with low mitotic activity and a small risk for malignant transformation. In contrast, medulloblastoma, which is the most common embryonic, invasive, solid tumor of the cerebellum, is classified as a WHO grade IV tumor (3). The therapeutic strategies and prognosis between these two tumor types are entirely different (1–3).

Histopathological assessment is the primary criterion for brain tumor diagnosis and grading. However, surgical biopsies are associated with morbidity and mortality risks and are not commonly accepted by all patients. The intraoperative error rate for microscopic diagnosis can reach as high as 8% (4). Therefore, precise diagnosis via neuroimaging is necessary prior to treatment initiation. When assessing posterior fossa tumors, brain magnetic resonance imaging (MRI) is the most popular diagnostic imaging technique, associated with certain unique radiological characteristics that can be used during the differential diagnosis. Unfortunately, pilocytic astrocytoma may appear as a less common, solid, non-necrotic tumor in as many as 10% of cases (5). Thus, because the radiological features associated with pilocytic astrocytoma are not always typical, an exact diagnosis cannot be efficiently performed using traditional methods (5–10).

Neoangiogenesis is defined as the formation of new blood vessels, derived from preexisting blood vessels and resulting from the creation of vascular chambers. This phase is essential for tumoral growth and the ability of tumors to metastasize. Tumor development consists of two stages. The first stage is considered the pre-angiogenesis period, during which the volume of the tumor does not change. The second stage includes tumoral growth, as a community of cells morphs into vascular-rich cells. The presence of vascular-rich tumoral cells causes an imbalance between angiogenic factors, such as basic fibroblast growth factor (bFGF) and vascular endothelial growth factor (VEGF), and anti-angiogenic factors, such as angiostatin. Neoangiogenesis enhances nourishment and oxygen supply, which are indispensable factors for tumoral growth (11–13).

The cerebral blood flow unit is calculated as ml/100 g/min, indicating the amount of blood (in milliliters) that circulates through 100 g of the brain parenchyma in 1 min. Currently, several techniques are available to assess cerebral perfusions, including nuclear imaging, computed tomography (CT) perfusion, dynamic susceptibility contrast (DSC) MRI, and arterial spin labeling (ASL). The primary advantage of ASL over nuclear imaging and CT is that the subject is not exposed to ionizing radiation during MRI. Furthermore, ASL is not based on material labeling, as in certain other methods, such as radionuclide agents or iodine/gadolinium-based contrast agents; therefore it is extremely beneficial for children, pregnancy, patients having severe allergic/anaphylactoid reaction to a contrast agent, and patients with eGFR below 30 mL/min/1.73 m^2^. Theoretically, water molecules are labeled and used to track blood flow in the brain, using a pulse generator. After a sufficient amount of time has passed to allow blood flow throughout the brain, a deduction procedure for the perfusion analysis is performed, using the “label” image relative to the “control” image, which is taken prior to labeling. Finally, ASL image can be generated quickly, offer valuable cerebral blood flow (CBF) details in <5 min (14, 15). Therefore, this study aimed to assess the use of three-dimensional (3D) pseudo-continuous ASL parameters to differentiate between pediatric medulloblastoma and pilocytic astrocytoma.

## Materials and Methods

### Subjects

The Institutional Review Board of Children's Hospital 02 approved this prospective study (Ref: 352/ND2-CÐT). Written informed consent was obtained from all patients' legal representatives prior to the MRI procedure. Inclusion criteria were: (1) between February 2019 and February 2020, (2) age <16 years, (3) preoperative MRI with ASL, (4) operated only at our institution, (5) histological diagnosis of medulloblastoma and pilocytic astrocytoma. Exclusion criteria included: (1) other tumor than medulloblastoma and pilocytic astrocytoma, (2) prior surgical treatment at a different institution, (3) previous interventions such as biopsy, stereotactic biopsy, and/or radiotherapy, (4) metastasis. Eventually, 33 patients were involved in this study, including 25 children diagnosed with medulloblastoma and eight children diagnosed with pilocytic astrocytoma.

### Anesthesia Procedure

All patients in this study were placed in a supine position on the MRI table. The physicians then administered midazolam (5 mg/1 ml) intravenously, at a dose of 0.1 mg/kg (Hameln Pharm GmbH, Germany), and 1% propofol (10 mg/1 ml), at a dose of 3 mg/kg (Fresofol, Fresenius Kabi GmbH, Austria), for anesthesia.

### MRI Procedure

All pediatric patients were scanned with 16-channel head coil in a 1.5 Tesla MRI machine (Multiva, Philips, Best, The Netherlands). All patients were studied using a 3D pseudo-continuous ASL sequence, with the following parameters: repetition time (TR): shortest; echo time (TE): shortest; flip angle: 90 degrees; slice thickness: 8 mm; gap: 0 mm; field of view: 240 × 140 mm; matrix: 64 × 60 mm; plane: axial; number of acquisition: 1; dynamics: 9 phases; post-labeling delay: 1,500 ms; and total duration: 4.44 min. The perfusion map was automatically derived from the 3D pseudo-continuous ASL sequence, after completing the scan.

### Investigative Parameters

**Figure 1:**
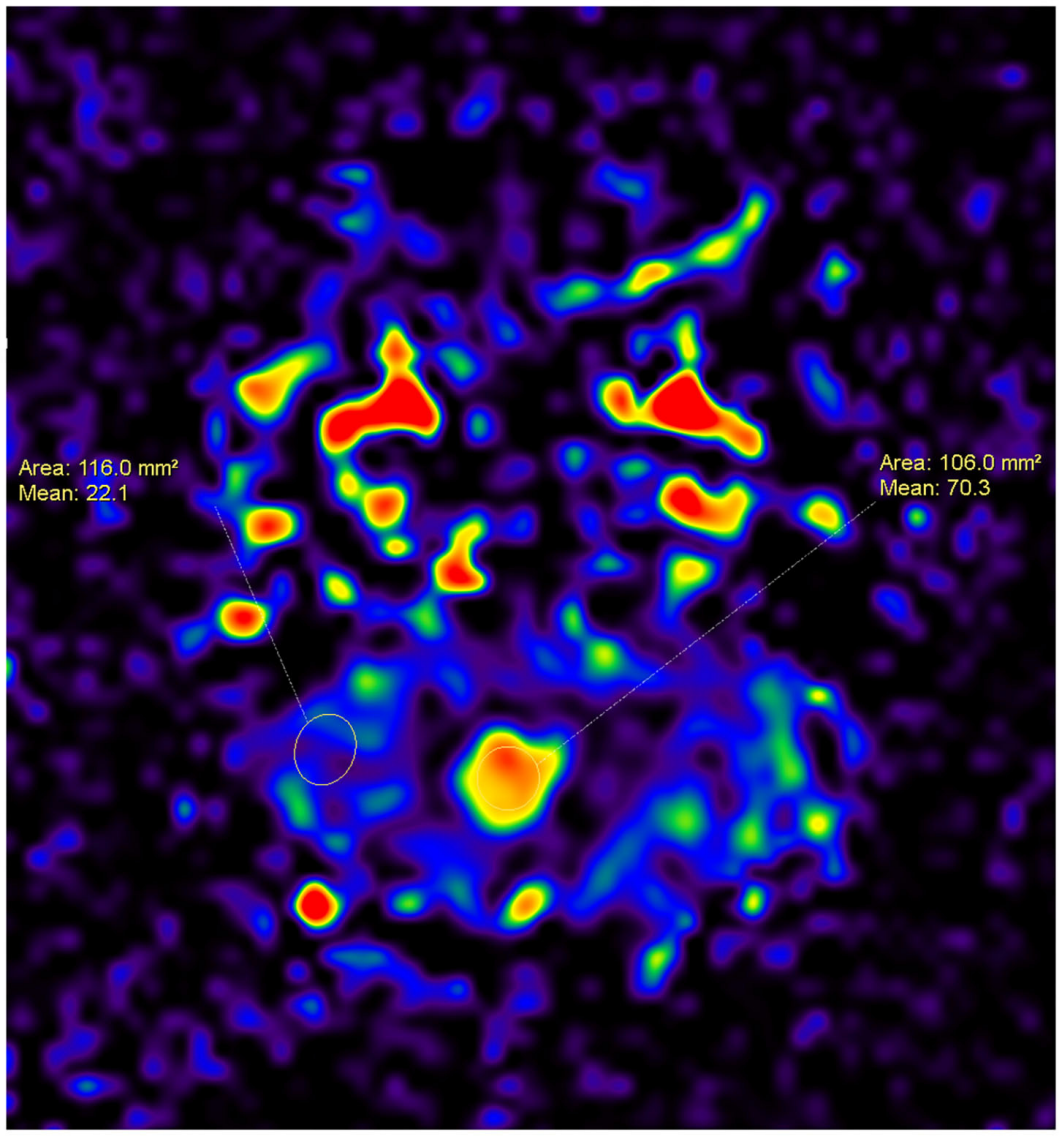
A 7-year-old female patient with a tumor inside the fourth ventricle, which was confirmed as medulloblastoma after surgery. An ASL map was created by drawing ROIs within the area of the tumor and the area of the parenchyma. The CBF for the medulloblastoma is higher than that for the parenchyma (70.3 vs. 22.1).

**Figure 2:**
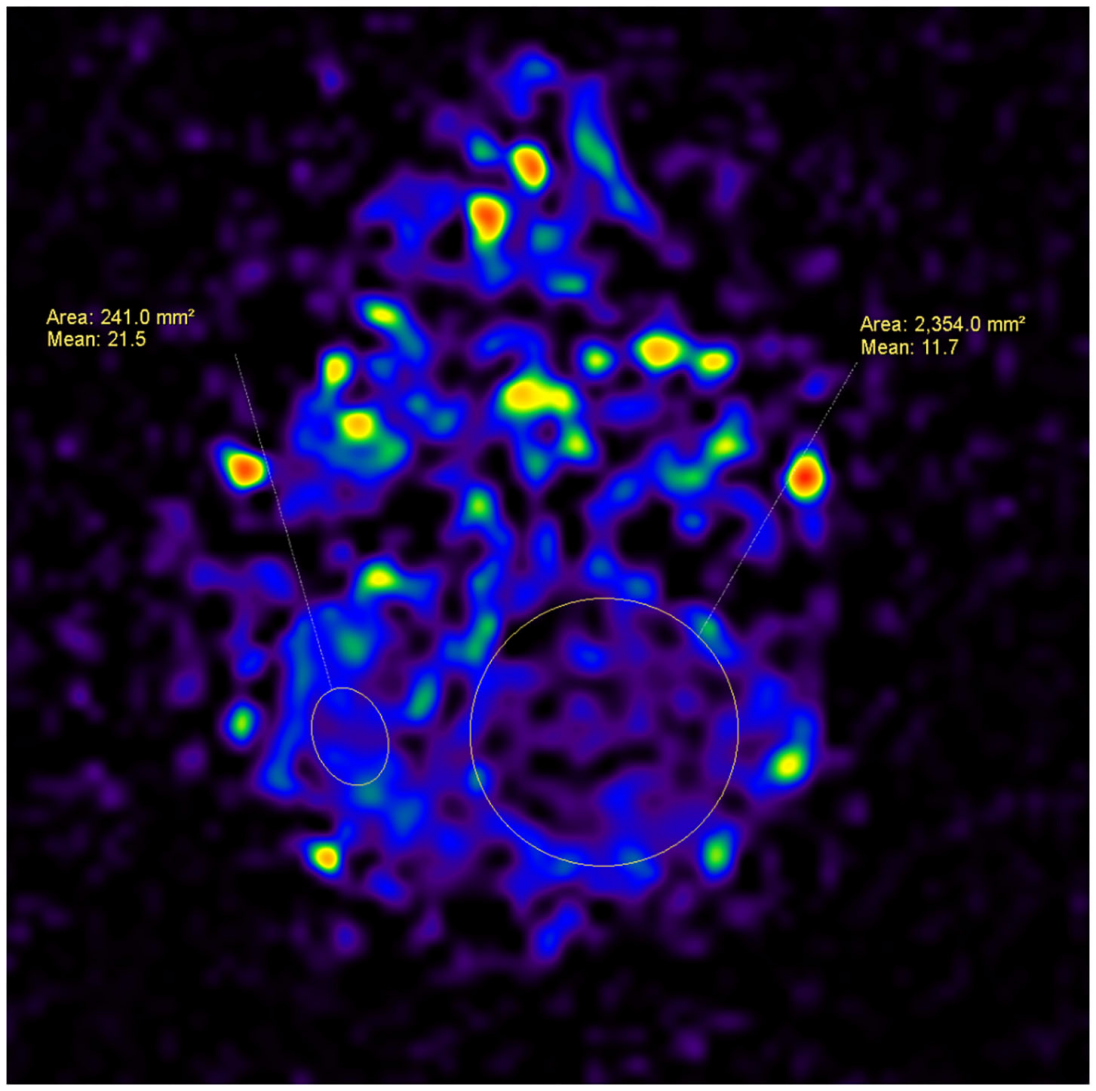
A 4-year-old male patient, with a tumor in the left cerebellar hemisphere, which was confirmed as pilocytic astrocytoma after surgery. An ASL map was analyzed by drawing ROIs within the area of the tumor and the area of the parenchyma. The CBF for the pilocytic astrocytoma is lower than that for the parenchyma (11.7 vs. 21.5).

The quantification of the perfusion map parameters was achieved by creating regions of interest (ROIs) in the tumor and parenchymal areas. The 3D pseudo-continuous ASL MRI data set was loaded onto a workstation (Philips, Best, The Netherland), and the CBF was automatically calculated. Based on the index from the perfusion map (Figures 1, 2), the tumor to parenchyma ratios was determined as the ratios between each tumoral CBF parameter and the corresponding parenchymal CBF parameter (rCBF).

### Statistical Analysis

SPSS software, version 26 (IBM Corp, Armonk, New York, USA) was used to perform statistical analysis. Non-normally distributed quantitative variables are presented as the median and interquartile range. We compared quantitative variables in this study using the Mann-Whitney U test. Receiver operating characteristic (ROC) curve analysis and the Youden index were used to assess the cut-off point, accuracy, sensitivity, and specificity of independent ASL parameters. Differences were considered to be significant when the *p*-value was <0.05.

## Results

Overall, the median age of population was 7 years and male/female ratio was 19/14. The median age of medulloblastomas and pilocytic astrocytomas was 8 and 6 years, respectively, and the male/female ratio of medulloblastomas and pilocytic astrocytomas was 14/11 and 5/3, respectively.

As shown in Table 1, the median CBF and rCBF of medulloblastoma were 16.06 and 0.96, respectively, meanwhile those of pilocytic astrocytoma were 9.28 and 0.41, respectively. CBF and rCBF values for medulloblastomas were significantly higher than those for pilocytic astrocytomas (*p* < 0.05).

**Table 1:** Comparison of ASL parameters between medulloblastoma and pilocytic astrocytoma.

**ASL parameters**	**Medulloblastoma**	**Pilocytic astrocytoma**	***P* value**
	**(*****n*** **=** **25)**	**(*****n*** **=** **8)**	
CBF	16.02 (39.00)	9.28 (10.05)	**0.019**^§^
rCBF	0.96 (1.95)	0.41 (0.37)	**0.005**^§^

*Data are presented as the median (interquartile range). ASL, arterial spin labeling; CBF, cerebral blood flow; rCBF, the ratio between the tumor and parenchyma CBF values*.

§ *Statistically significant*.

**Figure 3:**
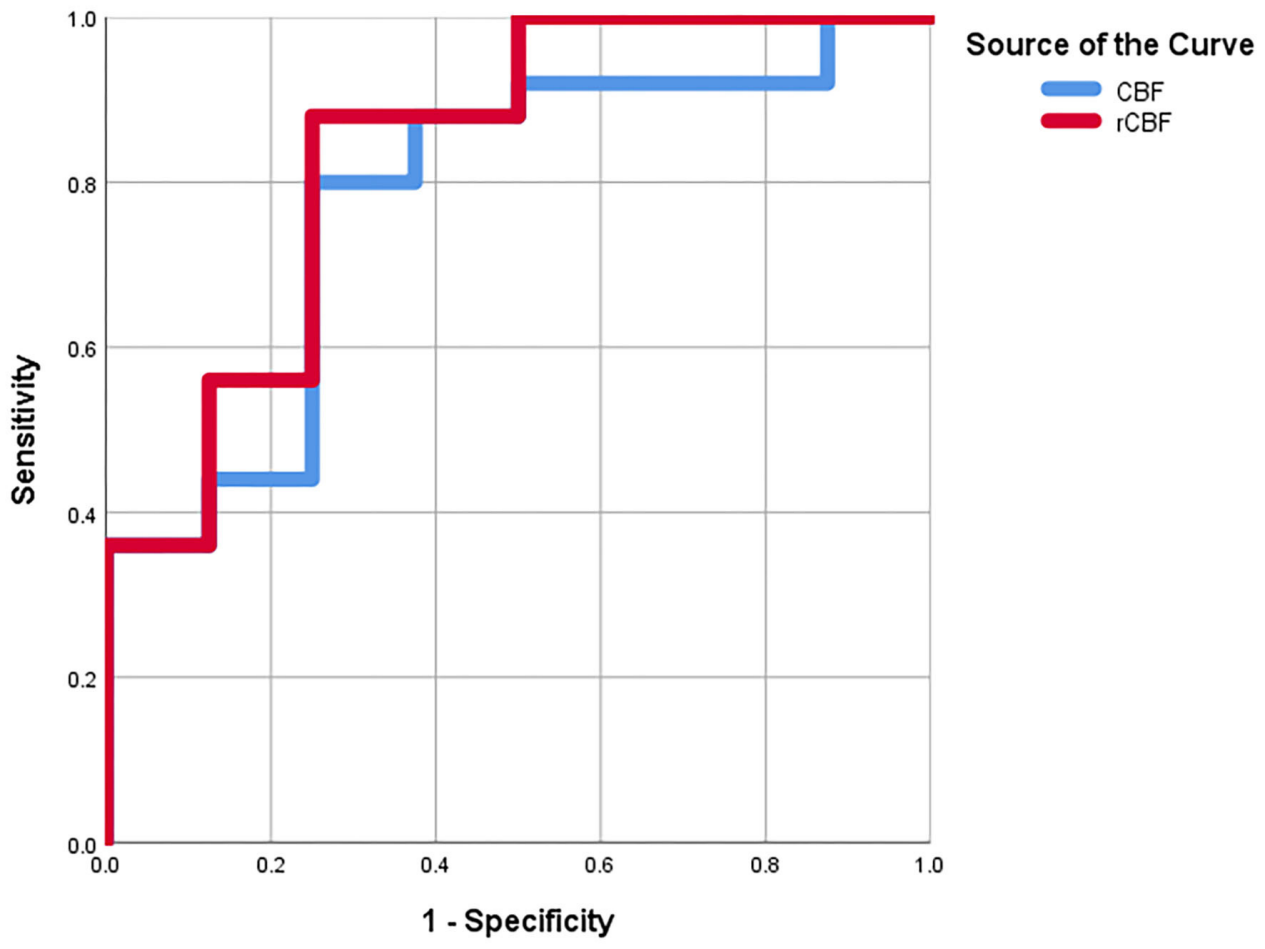
The receiver operating characteristic (ROC) curves for CBF and rCBF.

As shown in Table 2, a CBF cut-off value of 11.35 could discriminate between medulloblastoma and pilocytic astrocytoma, generating a sensitivity of 80%, a specificity of 75%, and an AUC of 78%. Meanwhile, an rCBF cut-off value of 0.51 could better differentiate between medulloblastoma and pilocytic astrocytoma, resulting in a sensitivity of 88%, a specificity of 75%, and an AUC of 83.5% (Figure 3).

**Table 2:** ROC analysis of ASL parameters for the differential diagnosis between medulloblastoma and pilocytic astrocytoma.

**ASL parameters**	**Cut-off point**	**AUC**	**Sensitivity**	**Specificity**	**95% CI**
CBF	11.35	0.780	0.8	0.75	0.594–0.966
rCBF	0.51	0.835	0.88	0.75	0.662–1.000

*ROC, receiver operating characteristic; ASL, arterial spin labeling; CBF, cerebral blood flow; rCBF, the ratio between the tumor and parenchyma CBF values; AUC, area under the ROC curve; CI, confidence interval*.

## Discussion

Tumoral neoangiogenesis is one of the most significant factors associated with the malignant diagnosis of glioma. The malignant glioma continues to exploit existing blood vessels, allowing them to regenerate, change shape, and create complex networks of blood vessels that provide further nutrients and oxygen to support tumoral growth and the ability to metastasize. Neovascularization also provides a pathway for tumor infiltration along vascular channels. Neovascularization also influences the distribution of drugs and the effectiveness of therapy or surgery. Chemotherapy is often suggested for highly malignant tumors, but not for tumors with low malignant potential. Therefore, the accurate diagnosis and grading of gliomas are crucial because these factors influence the decision-making process for treatment and prognosis (11–13).

ASL has been used to evaluate the penumbra area, such as during cerebral infarction, and has also been effective for assessing brain tumors and neurodegenerative disorders, such as Alzheimer's disease, dementia, and Parkinson's disease (16, 17). Recently, some studies have shown that ASL-measured CBF can differentiate between low- and high-grade astrocytomas (18, 19). In our study, the CBF and rCBF values for medulloblastomas compared with pilocytic astrocytomas were 16.02 vs. 9.28 and 0.96 vs. 0.41 (*p* < 0.05), respectively. Wang et al. studied 52 patients with glioma, divide into two groups: low-grade glioma (*n* = 16) and high-grade glioma (*n* = 36). Their results showed that maxCBF, rmaxCBF, meanCBF, and rmeanCBF values for the low-grade group were significantly lower than those for the high-grade group (20). A meta-analysis by Kong et al., including 305 glioma patients, also showed that the ASL perfusion values of the high-grade glioma group, including aCBFmax, aCBFmean, rCBFmax, and rCBFmean, were significantly higher than those of low-grade glioma group. In a previous report by Kong et al., the standardized mean difference values of aTBFmax, aTBFmean, rTBFmax, and rTBFmean between high-grade and low-grade gliomas were 0.70, 0.86, 1.08, and 0.88, respectively (*p* < 0.05). Kong et al. suggested that the tumoral CBF values arisen from ASL sequence could contribute substantially to glioma classification (19). Furthermore, Yeom et al. reported that in pediatric brain tumors, the rCBF values were significantly increased in grade-3 and−4 tumors (2.14 ± 1.78) compared with those in grade-1 and−2 tumors (0.60 ± 0.29) (*p* < 0.05). They also concluded that rCBF was significantly higher in medulloblastoma compared with pilocytic astrocytoma (*p* < 0.05) (21). Our findings were very consistent with these previous studies (18–21).

In the present study, among the examined ASL parameters, a cut-off rCBF value of 0.51 may differentiate medulloblastoma from pilocytic astrocytoma, with a sensitivity of 88%, a specificity of 75%, and an AUC of 83.5%. Wang et al. suggested an optimal cut-off rmaxCBF value of 1.25, which provided a sensitivity of 86.5%, a specificity of 73.3%, and an AUC of 79.8%, whereas an optimal cut-off rmeanCBF value of 0.89 yielded a sensitivity of 70.3%, a specificity of 86.7%, and an AUC of 74.4%. The rmaxCBF value showed the best diagnostic efficacy, whereas the rmeanCBF value had the best specificity for grade discrimination (20). In a meta-analysis examining 15 previous studies, involving 505 glioma patients, Falk Delgado et al. found that the diagnostic performance of CBF values for glioma grading was 0.90, with a sensitivity of 0.89 (0.79–0.90) and a specificity of 0.80 (0.72–0.89). They also concluded that CBF values had excellent diagnostic accuracy for the differentiation between high-grade and low-grade gliomas owing to cost-effectiveness, lack of adverse events, and high acceptibility (18). Thus, our findings were in agreement with these previous reports (18, 20).

Pilocytic astrocytoma, a grade I tumor according to the WHO classification, is generally found in the cerebellum. These tumors are more common in children than in adults. Such tumors are easily recognizable on histopathological assessment due to the appearance of hairy stromal cells or astrocytes. Rosenthal fibers and microcystic alterations are also observed. Pilocytic astrocytomas are composed of only a small number of cells, with weak cellular proliferation and mitosis, and this type of tumor rarely invades the surrounding tissue or metastasizes. Conversely, medulloblastoma can be oval or round and primarily consists of small cells, with minimal cytoplasm and homogeneous, dark nuclei. Medulloblastoma is a very malignant tumor, distinguished by increased cell density and rapid cell division. It is graded as grade IV, which represents the highest malignant potential according to the WHO classification. Malignant tumors display increased angiogenesis compared with lower-grade tumors, due to the upregulation of proangiogenic factors (VEGF and bFGF) and the downregulation of inhibitors (interferon and angiostatin). Increased angiogenesis is the primary explanation for why perfusion differs between medulloblastoma and pilocytic astrocytoma (4–6, 8, 18–26).

There are several disadvantages of ASL. Firstly, ASL signal-to-noise is usually low in comparison with DSC (27). In addition, due to the requirement to generate tag and control images and to support time for blood to be transferred from the tagging area to the monitoring slice, the ASL temporal resolution is fundamentally low. One tag and control image pair is acquired approximately every 4 s in a standard pseudo-continuous ASL experiment. Furthermore, ASL always has less coverage (3–15 slices with thickness of slices extending from 5 to 8 mm) (28, 29).

## Limitations

The small sample size and the single-center involvement associated with the present study could be viewed as drawbacks for this study. Furthermore, this study recruited a comparatively smaller number of pilocytic astrocytoma patients. We have only compared two types of tumors in this study. We also focused on the role of 3D pseudo-continuous ASL in separating medulloblastomas from pilocytic astrocytomas; thus, lacking the information and investigation of conventional sequences. It is noted that we also did not include medulloblastomas with metastasis in this study and 3D pseudo-continuous ASL cannot replace T1-weighted imaging with contrast enhancement in the evaluation of medulloblastomas with metastasis. We propose that further studies be conducted to confirm our results, utilizing broader sample sizes, a multicenter design, and the assessment of more various tumor types such as ependymoma and atypical teratoid/rhabdoid tumor. In addition, in comparison with diffusion-weighted imaging (DWI), 3D pseudo-continuous ASL is more time-consuming, complex, and unpopular. For improving the discrimination among pediatric posterior fossa brain tumors, the combination of 3D pseudo-continuous ASL and DWI is very essential to be carried out in the future research.

## Conclusion

To sum up, our study recommends that the CBF and rCBF values, derived from 3D pseudo-continuous ASL, could be served as important differentiating factors between pediatric medulloblastoma and pilocytic astrocytoma. Among these parameters, a cut-off value of 0.51 for rCBF was effective for the discrimination between medulloblastoma and pilocytic astrocytoma. During the daily workup of pediatric patients with medulloblastoma or pilocytic astrocytoma, ASL should be considered a supplemental test, due to its non-invasiveness, the avoidance of ionizing radiation, contrast-free quantitative CBF, consistent over time, less complex across subjects, and the effective diagnostic differentiation.

## Data Availability Statement

The original contributions presented in the study are included in the article/supplementary material, further inquiries can be directed to the corresponding author/s.

## Ethics Statement

The studies involving human participants were reviewed and approved by the institutional review board of Children's hospital 2 approved this prospective study (Ref: 352/ND2-CÐT). Written informed consent to participate in this study was provided by the participants' legal guardian/next of kin.

## Author Contributions

The author confirms being the sole contributor of this work and has approved it for publication.

## Conflict of Interest

The author declares that the research was conducted in the absence of any commercial or financial relationships that could be construed as a potential conflict of interest.
